# Supplementary material for: A multivariate Box-Behnken assessment of elevated branched-chain amino acid concentrations in reduced crude protein diets offered to male broiler chickens
Source: PLoS One. 2022 Mar 30;17(3):e0266080. doi: 10.1371/journal.pone.0266080 (PMC8967063; doi:10.1371/journal.pone.0266080)
Supplement: Attachment — Submitted filename: Response_to_reviewers.docx [file pone.0266080.s002.docx]

Poultry Research Foundation

within The University of Sydney

Thursday, 29^th^ July, 2021

Editorial Office

Plos One

Dear Dr Juan J Loor

On behalf of my co-authors, I would like to submit the revision of PONE-D-21-12314,

‘A multivariate Box-Behnken assessment of elevated branched-chain amino acid concentrations in reduced crude protein diets offered to male broiler chickens’

Please see the below updates as required

**Funding Statement**

The study is funded by Evonik Nutrition and Care GmbH, Hanau-Wolfgang 63457, Germany. The funder provided support in the form of salaries for authors Dr Juliano C. de P Dorigam, but did not have any additional role in the study design, data collection and analysis, decision to publish or preparation of the manuscript. The specific roles of these authors are articulated in the ‘author contributions’ section.

**Competing Interests Statement**

The study is funded by Evonik Nutrition and Care GmbH, Hanau-Wolfgang 63457, Germany. However, this does not alter our adherence to PLOS ONE policies on sharing data and materials. Dr Juliano C. de P Dorigam is employed by Evonik and he contributed to Funding acquisition and Project administration.

Thank you for reviewers’ suggestions and our detailed response is attached in the Appendix. We look forward to hearing from you and the reviewers.

Yours Sincerely

Dr Sonia Y. Liu

Senior Lecturer in Poultry Nutrition

Poultry Research Foundation

**Appendix**

Journal Requirements:

When submitting your revision, we need you to address these additional requirements.

1. Please ensure that your manuscript meets PLOS ONE's style requirements, including those for file naming. The PLOS ONE style templates can be found at [https://journals.plos.org/plosone/s/file?id=wjVg/PLOSOne_formatting_sample_main_body.pdf](https://protect-au.mimecast.com/s/GckrCK1DvKT2Lz9NrfAQc7c?domain=journals.plos.org) and [https://journals.plos.org/plosone/s/file?id=ba62/PLOSOne_formatting_sample_title_authors_affiliations.pdf](about:blank)

Checked and corrected

2. Please include captions for your Supporting Information files at the end of your manuscript, and update any in-text citations to match accordingly. Please see our Supporting Information guidelines for more information: [http://journals.plos.org/plosone/s/supporting-information](about:blank).

Included

3.  Thank you for stating the following in the Financial Disclosure section:

'The study is funded by Evonik Nutrition and Care GmbH, Hanau-Wolfgang 63457, Germany'

We note that you received funding from a commercial source: Evonik Nutrition and Care GmbH

Please provide an amended Competing Interests Statement that explicitly states this commercial funder, along with any other [relevant declarations](about:blank) relating to employment, consultancy, patents, products in development, marketed products, etc.

Within this Competing Interests Statement, please confirm that this does not alter your adherence to all PLOS ONE policies on sharing data and materials by including the following statement: "This does not alter our adherence to PLOS ONE policies on sharing data and materials.” (as detailed online in our guide for authors [http://journals.plos.org/plosone/s/competing-interests](about:blank)).  If there are restrictions on sharing of data and/or materials, please state these. Please note that we cannot proceed with consideration of your article until this information has been declared.

Please include your amended Competing Interests Statement within your cover letter. We will change the online submission form on your behalf.

Updated

4. Thank you for stating the following in the Competing Interests section:

'The authors have no conflicts of interest to declare.'

We note that one or more of the authors are employed by a commercial company: Baiada Poultry Pty Limited and Evonik Operations GmbH.

1. Please provide an amended Funding Statement declaring this commercial affiliation, as well as a statement regarding the Role of Funders in your study. If the funding organization did not play a role in the study design, data collection and analysis, decision to publish, or preparation of the manuscript and only provided financial support in the form of authors' salaries and/or research materials, please review your statements relating to the author contributions, and ensure you have specifically and accurately indicated the role(s) that these authors had in your study. You can update author roles in the Author Contributions section of the online submission form.

Please also include the following statement within your amended Funding Statement.

“The funder provided support in the form of salaries for authors [insert relevant initials], but did not have any additional role in the study design, data collection and analysis, decision to publish, or preparation of the manuscript. The specific roles of these authors are articulated in the ‘author contributions’ section.”

If your commercial affiliation did play a role in your study, please state and explain this role within your updated Funding Statement.

Updated

2. Please also provide an updated Competing Interests Statement declaring this commercial affiliation along with any other relevant declarations relating to employment, consultancy, patents, products in development, or marketed products, etc.

Within your Competing Interests Statement, please confirm that this commercial affiliation does not alter your adherence to all PLOS ONE policies on sharing data and materials by including the following statement: "This does not alter our adherence to  PLOS ONE policies on sharing data and materials.” (as detailed online in our guide for authors [http://journals.plos.org/plosone/s/competing-interests](about:blank)) . If this adherence statement is not accurate and there are restrictions on sharing of data and/or materials, please state these. Please note that we cannot proceed with consideration of your article until this information has been declared.

Please include both an updated Funding Statement and Competing Interests Statement in your cover letter. We will change the online submission form on your behalf.

Please know it is PLOS ONE policy for corresponding authors to declare, on behalf of all authors, all potential competing interests for the purposes of transparency. PLOS defines a competing interest as anything that interferes with, or could reasonably be perceived as interfering with, the full and objective presentation, peer review, editorial decision-making, or publication of research or non-research articles submitted to one of the journals. Competing interests can be financial or non-financial, professional, or personal. Competing interests can arise in relationship to an organization or another person. Please follow this link to our website for more details on competing interests: [http://journals.plos.org/plosone/s/competing-interests](about:blank)

Updated

**Comments to the Author**

1. Is the manuscript technically sound, and do the data support the conclusions?

The manuscript must describe a technically sound piece of scientific research with data that supports the conclusions. Experiments must have been conducted rigorously, with appropriate controls, replication, and sample sizes. The conclusions must be drawn appropriately based on the data presented.

Reviewer #1: No

The Box-Behnken design (BBD) in nutrition research is largely based on de Leon et al., described in reference # [24] of the submitted manuscript. These authors applied a BBD investigating the responses of male broilers to lysine, threonine and total sulphur amino acids from 15 to 35 days post-hatch. In their study, treatments were replicated 5 times based on Myers and Montgomery (1995) recommendations. For this reason, we selected the 5 repeats as being sufficient for a BBD to demonstrate required responses/antagonisms with branched chain amino acids.

2. Has the statistical analysis been performed appropriately and rigorously?

Reviewer #1: No

Agree with the reviewer - In retrospect, a simple one-way ANOVA is not the correct way to do response surface methodology (RSM) and the statistics have been re-run as a BBD in JMP Pro version 15.2.0. The methodology describes externally studentized residuals with 95% simultaneous limits (Bonferroni).

3. Have the authors made all data underlying the findings in their manuscript fully available?

The [PLOS Data policy](https://protect-au.mimecast.com/s/c5VOCOMKzVTp3zwj2tjpHrH?domain=plosone.org) requires authors to make all data underlying the findings described in their manuscript fully available without restriction, with rare exception (please refer to the Data Availability Statement in the manuscript PDF file). The data should be provided as part of the manuscript or its supporting information, or deposited to a public repository. For example, in addition to summary statistics, the data points behind means, medians and variance measures should be available. If there are restrictions on publicly sharing data—e.g. participant privacy or use of data from a third party—those must be specified.

Reviewer #1: Yes

4. Is the manuscript presented in an intelligible fashion and written in standard English?

PLOS ONE does not copyedit accepted manuscripts, so the language in submitted articles must be clear, correct, and unambiguous. Any typographical or grammatical errors should be corrected at revision, so please note any specific errors here.

Reviewer #1: Yes

5. Review Comments to the Author

Please use the space provided to explain your answers to the questions above. You may also include additional comments for the author, including concerns about dual publication, research ethics, or publication ethics. (Please upload your review as an attachment if it exceeds 20,000 characters)

Reviewer #1: This manuscript is a potential addition to several recent papers attempting to better understand branched-chain amino acid (Val, Ile, and Leu) utilization, specifically in the context of reduced protein diets. This is a well-organized and written manuscript with minimal to no technical or grammatical errors. However, there are several major concerns regarding the design of the experiment.

1) Dietary amino acid (BCAA) levels tested: The recommended dietary concentrations of Val, Ile, and Leu for Ross 308 broiler for this age (per Aviagen specifications) are 0.87, 0.78, and 1.27%, respectively, with a Lys recommendation of 1.15%. Thus, ratios relative to Lys are 76, 68, and 110 for Val, Ile, and Leu. In this experiment (Table 1) Val:Lys ratios evaluated ranged from 89 to 125 (17 to 64% greater than recc.), Ile:Lys ratios ranged from 68 to 109 (6 to 60% greater than recc.), and Leu:Lys ratios ranged from 125 to 183 (14 to 66% greater than recc.).
Why were the lowest levels of each BCAA tested 6 to 17% higher than current recommendations? From a practical standpoint, least-cost formulation will dictate that digestible BCAA are at the minimum level specified, especially in reduced CP diets, and this specified minimum will likely be similar to current recommendations. From a scientific perspective, the conclusion of “…when individual BCAA are not deficient, they will not result in antagonism” (line 427) should be changed to “…when BCAA are in marked excess of current recommendations…” The practical or scientific merit of such a conclusion is not clear.

Normal grower diets based on maize/soyabean meal never minimize on digestible leucine. Typically, these diets have a ratio of digestible leucine: digestible lysine greater than 1.25:1. If one uses DDGS, then the ratio widens further in standard diets. Since this trial was aimed at testing branched chain amino acid (BCAA) antagonism, the starting point was selection of a typical dietary leucine level, even though, as dietary CP declines, the minimum ratios of all BCAA:Lys tend to be approached. The trial was designed specifically to test high levels of BCAA on broiler growth performance, digestive dynamics and blood plasma profiles. Thus the initial BCAA levels were chosen to exceed the primary breeder requirements. The wide range of BCAA ratios tested should have resulted in substantial antagonisms of BCAA or responses to BCAA – if they exist in reduced protein diets.

The conclusion was changed as suggested.

2) Formulation approach: The authors clearly took great care to balance the diets. However, the approach for doing so resulted in considerable changes in what should be a relatively constant “basal” component of the diet. For example, soy oil, which can have effects independent of calorie contribution, ranged from 2.77 to 11.1 g/kg (4x and additional ~75 kcal contribution from oil), and corn and SBM inclusions varied as well. Alternatively, BCAA could have been varied at the expense of glutamic acid and corn starch, likely allowing isonitrogenous, isocaloric, and balanced digestible AA with constant corn and SBM inclusions and only minor variations in soy oil.

The diets were deliberately formulated to mimic commercial-type diets so corn starch was avoided. However, the AME of glutamic acid is about half that of the BCAA and maize starch about two-thirds, so one cannot get iso-energetic diets with that combination. The crude protein matrix values used for “as fed” are 595 g/kg for glutamic acid and, 732.3, 640.0 and 657.0 g/kg for Val, Ile and Leu respectively so, once again it is difficult to get iso-nitrogenous diets with an amino acid that has a lower N content than the test BCAA. Also, there is some evidence to suggest that glutamic acid itself could confound the trial. Previous work on BCAA often excludes a CP value for the test AA and then simply utilise an inert filler. In our view, this changes the fundamental balance of the diet since dietary CP would change as would AME by treatment. This would therefore confound the effects of just the 3 BCAA levels being tested.

As far as dietary added oil is concerned, the authors note that all diets contained canola seed at 60 g/kg supplying ~ 25 g/kg oil, so the soya oil is simply added as a top-up to balance the dietary AME and ensure these diets are all iso-caloric. The total crude fat of the diets ranged from 59.0 to 67.4 g/kg (treatments 5 & 12) representing a difference of 14.2%. Once again, this would not be seen as excessive and is well within normal commercial dietary fat inclusions. Interestingly, these two treatments had almost identical measured AME of 13.39 and 13.37 MJ/kg respectively (13.38 and 13.39 MJ/kg AME_n_) so no evidence of extra-caloric effects. The widest range of measured AME was 13.07 to 13.49 MJ/kg (treatments 2 and 7 respectively) yet these diets were similar at 62.6 and 62.0 g/kg total crude fat.

Based on data from Leeson & summers (2005) and van der Klis (2010), the relative energy change is approximately 2% for a change from 59 to 67 g/kg added fat to a diet. So, if soya oil is used at 37 MJ/kg and a 2% decline is used, this will drop to 36.26 MJ/kg at the higher inclusion level. The difference in dietary energy at 67 g/kg inclusion is therefore over-estimated by 0.05 MJ/kg (2.479 versus 2.429 MJ/kg) or 12 kcal/kg feed and this is marginal in our view. The AME system has a number of limitations anyway and it is unclear what the “extra-caloric” values for the small volumes of added soya oil that are questioned could be?

The small changes in SBM, maize and oil were thus necessary in our view and somewhat novel to other reported work as referenced in the manuscript.

3) Statistical analysis: The reviewer understands that the high number of treatments required in this design limits capacity for replication. Five replications are low but likely acceptable for the primary objectives of dose response evaluation. However, this replication was likely too low for making means comparisons. Further, the method for separating means should be stated (Tukey’s or other adjustment necessary), and if it was Fisher’s LSD (assumption based on LSD value reported in table), this would be inappropriate given the large number of treatments. Much of the results and conclusions are mean comparisons, however, lack of replication and use of unadjusted mean comparison test are concerning for Type 2 and Type 1 errors, respectively. This reviewer believes that interpretation should only be based on independent and interactive linear and quadratic dose responses and not individual means comparisons.

As noted for reviewer #1 - The Box-Behnken design (BBD) in nutrition research is largely based on de Leon et al., described in reference # [24] of the submitted manuscript. These authors applied a BBD investigating the responses of male broilers to lysine, threonine and total sulphur amino acids from 15 to 35 days post-hatch. In their study, treatments were replicated 5 times based on Myers and Montgomery (1995) recommendations. For this reason, we selected the 5 repeats as being sufficient for a BBD to demonstrate required responses/antagonisms with branched chain amino acids.

Additionally, in retrospect, a simple one-way ANOVA is not the correct way to do response surface methodology (RSM) and the statistics have been re-run as a BBD in JMP Pro version 15.2.0.

In summary, to be suitable for publication, this paper should be revised to bring forward and provide reasonable justification for use of such high levels of BCAA (5 to 60% excess of likely requirement), and data interpretation should be based only on dose responses. Given that primary ingredients outside of those tested varied across treatments, responses on dietary AME, N utilization, and AA digestibility should be deemphasized.

In a series of trials in our laboratory, digestive dynamics including AME, N utilisation and AA digestibility have been included. Our contention is that this is important in elucidating treatment effects at the nutrient and metabolic levels. However, we are cognisant that this section can be de-emphasised and will do so.

The justification for using extreme levels of BCAA in this trial was to ensure that antagonisms reported in the literature could be investigated without having to extrapolate the data beyond the experimental treatments, which is one of the potential pitfalls of RSM. If a dose response existed for BCAA antagonisms, using ratios that were high, were worth investigating in our view. This was novel for this study only because it was a 3-factor BBD. In recent work, Zeitz et al., (2019) investigated ratios of 35 to 60% above the breeder recommendations (references #20 & #21 in the manuscript) so it isn’t completely unusual to test such wide ratios. It would appear that antagonistic effects may only happen in atypical diets that are marginal in Ile and Val.
