# Supplementary material for: A multivariate Box-Behnken assessment of elevated branched-chain amino acid concentrations in reduced crude protein diets offered to male broiler chickens
Source: PLoS One. 2022 Mar 30;17(3):e0266080. doi: 10.1371/journal.pone.0266080 (PMC8967063; doi:10.1371/journal.pone.0266080)
Supplement: Attachment — Submitted filename: Response_to_reviewers_R2.docx [file pone.0266080.s003.docx]

**Appendix**

Reviewer #2: General comments:

This work represents a BCAA BB experiment to assess levels of BCAA in adequacy to excessive level cross product interactions. Indeed, more work with BCAA is needed in broiler nutrition.

The corrections in R1 to remove mean comparisons are very important, as BB designs allow for response surface cross product trend responses, not for the comparison of predicted means (Tables 6 onward are now valid). Some scientists disagree with means being presented in BB studies, just response curves, but I think providing the data allow for future modeling studies. However, why were p values maintained on Tables 15-17, these should be removed?

P-values are removed from tables 15-17

In general, a lot of the discussion focuses on individual predicted treatments, which is not the intended outcome of the experimental design. Tailoring the discussion to the general trends based on the generated response surfaces or linear and quadratic trends will improve the paper.

Specific comments

L103-114 in R1 I feel more explanation of the experimental design treatment levels is needed. The argument of branched-chain amino acid antagonism being a major issue in reduced CP diets is not synonymous in BCAA excesses, nor industry relevance when feeding reduced CP. Some sort of digestive hypothesis needs to be attempted. For example, and just an example… “Hence, there have been studies conducted testing isoleucine and valine levels at practical levels when leucine is in excess, therefore we tested excess levels of all three in an attempt to determine if allowing for a “balanced” internal ratio allows for correction of the negative effects of leucine.”

It now reads like this,

BCAA. The removal of supplemented Val from a balanced reduced crude protein diet caused the largest reduction on growth performance in comparison to removal of others supplemented amino acids and the removal of Leu significantly increased Val and Ile concentrations in plasma [24]. Previous studies examined moderate ranges of BCAA derived from formulating conventional diets [25, 26] and it is hypothesised the importance of BCAA may be more pronouced in reduced CP diets, hence the purpose

In Table 8 there is a column of cross-product p vales, this should be footnoted as to the effect. As BB designs provide three cross-product responses. I feel more explanation to the fitted model is needed. Same in Table 9, footnotes need to be added to explain the data.

The cross-product columns are deleted in both tables 8 and 12. Footnote is not included because Tables 9 and 13 included the significance of each cross-product.

L194 Why were only the highest Leu used? And the last treatment does not match Table 15 (10J or 11K). And I feel in all cases of treatment coding (5E-10J and 1-13) in tables, that the actual BCAA levels should be listed to aid in reader interpretation.

The below is included

The removal of supplemented Leu increased Val and Ile levels in plasma; whereas the removal of Val and Ile individually did not alter BCAA concentrations in plasma [24]. Therefore, at 27 days post-hatch, three birds at random were selected from each cage of the highest leucine treatments (5E, 7G, 9I and 11K)

L499 in R1, both Maynard et al. [29] and Kidd et al [28] had BCAA carcass products interactions.

Corrected
